# Random Single Amino Acid Deletion Sampling Unveils Structural Tolerance and the Benefits of Helical Registry Shift on GFP Folding and Structure

**DOI:** 10.1016/j.str.2014.03.014

**Published:** 2014-06-10

**Authors:** James A.J. Arpino, Samuel C. Reddington, Lisa M. Halliwell, Pierre J. Rizkallah, D. Dafydd Jones

**Affiliations:** 1 School of Biosciences, Main Building, Park Place, Cardiff University, Cardiff CF10 3AT, UK; 2 School of Medicine, Cardiff University, WHRI, Main Building, Heath Park, Cardiff CF14 4XN, UK

## Abstract

Altering a protein’s backbone through amino acid deletion is a common evolutionary mutational mechanism, but is generally ignored during protein engineering primarily because its effect on the folding-structure-function relationship is difficult to predict. Using directed evolution, enhanced green fluorescent protein (EGFP) was observed to tolerate residue deletion across the breadth of the protein, particularly within short and long loops, helical elements, and at the termini of strands. A variant with G4 removed from a helix (EGFP^G4Δ^) conferred significantly higher cellular fluorescence. Folding analysis revealed that EGFP^G4Δ^ retained more structure upon unfolding and refolded with almost 100% efficiency but at the expense of thermodynamic stability. The EGFP^G4Δ^ structure revealed that G4 deletion caused a beneficial helical registry shift resulting in a new polar interaction network, which potentially stabilizes a *cis* proline peptide bond and links secondary structure elements. Thus, deletion mutations and registry shifts can enhance proteins through structural rearrangements not possible by substitution mutations alone.

## Highlights

• Using directed evolution, the impact of amino acid deletion on EGFP is explored
• Loops, helices, and strand termini are especially tolerant to amino acid deletion
• A deletion mutant that enhances cellular production and fluorescence is identified
• Structure reveals that a helical registry shift creates a new polar network

## Graphical Abstract

**Figure:**
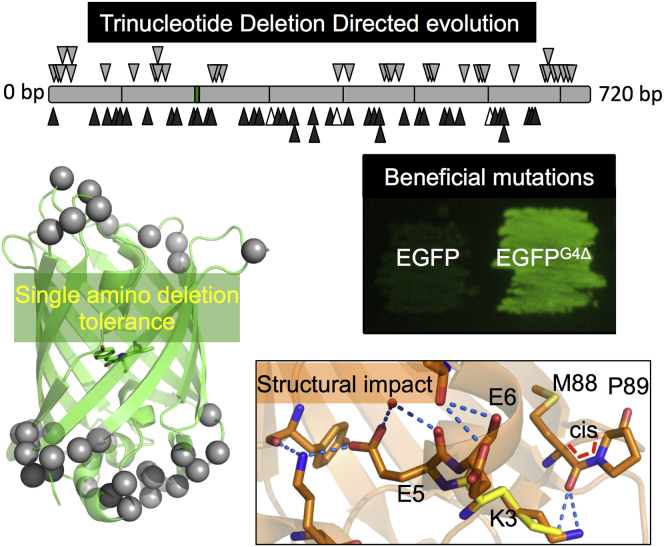

## Teaser

Using directed evolution, Arpino et al. examine the impact of amino acid deletion on EGFP and find that loops, helices, and strand termini are especially tolerant to amino acid deletion. Structural work provides a molecular explanation for this observation.

## Introduction

Protein backbone mutations or amino acid insertion/deletion (InDel) events are an important part of the natural evolutionary process (de Jong and Rydén, 1981; Leushkin et al., 2012; Taylor et al., 2004; Tóth-Petróczy and Tawfik, 2013) and affect protein structure in a manner distinct to that of side chain substitution (Pascarella and Argos, 1992; Shortle and Sondek, 1995). InDels are now thought to be key contributors to the evolutionary process by instigating major leaps in the protein fitness landscape (Leushkin et al., 2012; Tóth-Petróczy and Tawfik, 2013). Thus, InDels provide a new route to increase sequence and structure sampling space during the protein engineering process (Shortle and Sondek, 1995). However, whether site-directed, computationally or directed evolution-driven, the main focus of protein engineering is the generation of amino acid substitutions. The absence of InDel mutagenesis as part of the routine protein engineering toolbox is partly due to the difficultly in predicting the local and global structural influence of altering the protein backbone; dogma suggests such mutations are likely to be detrimental due to, for example, disruptive registry shifts in organized secondary structure and perturbing folding pathways (Pascarella and Argos, 1992; Shortle and Sondek, 1995). Consequently, there have been relatively few studies concerning the structural impact of engineered InDel mutations (Arpino et al., 2012a; Heinz et al., 1993; O’Neil et al., 2000; Stott et al., 2009; Vetter et al., 1996), especially regarding how any beneficial effects are exerted at the molecular level (Arpino et al., 2012a). Most of these studies have focused on site-directed introduction of InDels, so little information is available on the general tolerance of proteins to InDels and their beneficial effect.

One of the most common backbone mutations observed among protein homologs is deletion of single amino acids (de Jong and Rydén, 1981; Taylor et al., 2004), leading to, for example, expansion of the antibody repertoire (de Wildt et al., 1999), emergence of HIV drug resistance (Imamichi et al., 2001; Wood et al., 2009), herbicide resistance (Patzoldt et al., 2006), and resistance to third-generation β-lactam antibiotics (Jones, 2005; Simm et al., 2007). The potential benefits of sampling single amino acid deletions as part of protein engineering endeavors has recently emerged predominately through the advent of directed-evolution approaches (Bershtein and Tawfik, 2008). Such directed-evolution approaches that sample single amino acid deletions (Fujii et al., 2006; Jones, 2005; Murakami et al., 2002) has allowed broader sampling across the whole protein backbone, which removes any perceived bias concerning tolerance and impact on the target protein. This in turn allows retrospective structural analysis to understand the molecular basis of action.

Here, we have applied a transposon-mediated directed-evolution trinucleotide deletion (TND) approach (Jones, 2005; Simm et al., 2007) to the commercially important and widely used enhanced green fluorescent protein (EGFP), an engineered variant of the original *Aequorea victoria* GFP (Tsien, 1998). EGFP is an important tool in cell biology (Tsien, 1998; Zhang et al., 2002) but still has some limitations resulting in further protein engineering endeavors to improve properties such as stability, folding efficiency, and solubility during cellular expression. Despite their relatively high thermodynamic stability (Tsien, 1998), GFPs suffer from off-pathway aggregation due to their slow folding and maturation times (Fukuda et al., 2000). The transposon-based tool has been used previously to sample various domain insertion and codon replacement events relating to EGFP (Arpino et al., 2012a; Baldwin et al., 2009) but without a thorough analysis of the impact of TNDs. Through the construction and screening of a TND library, the general tolerance and impact of single amino acid deletions were explored. A variant with a single amino acid deletion was identified that conferred a brighter fluorescence phenotype on *Escherichia coli*. Rather than altering the spectral characteristics, the deletion mutation caused local structural rearrangements in a 3_10_ helix, resulting in new long-range polar interactions, including with the sole *cis* proline peptide bond.

## Results

### Tolerance of EGFP to Single Amino Acid Deletion

**Figure 1:**
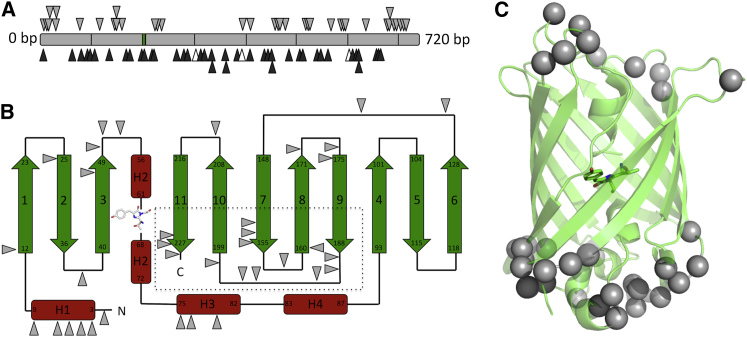
Mapping Deletion Mutations with Respect to EGFP Primary, Secondary, and Tertiary Structure (A) Gene sequence analysis of fluorescent (gray triangles) and nonfluorescent variants (black triangles) selected during the screening process identified the position of the triplet nucleotide deleted from *egfp* (gray bar). Nonfluorescent variants due to a TND and subsequent introduction of a premature stop codon are highlighted by white triangles. (B) The secondary structure arrangement and overall topology of EGFP shows the arrangement of β strands (green), α helices (red), and loops (black). Tolerated single amino acid deletions are indicated by gray triangles with an area particularly tolerant to deletion mutations surrounded by a dotted line. (C) Map of single amino acid deletions onto the tertiary structure of EGFP. Cartoon representation of EGFP (green) with tolerated deletions indicated by gray spheres.

The EGFP TND library was constructed essentially as described elsewhere (Baldwin et al., 2009; Simm et al., 2007) and screened for a green fluorescence *E*. *coli* phenotype upon irradiation with near UV light. Only correctly folded EGFP variants bestow the fluorescent green phenotype on *E*. *coli*. Of all the colonies screened, 10% displayed green fluorescence after extended growth, with 2.5% displaying noticeable green fluorescence after 24 hr at 37°C. A total of 153 colonies were chosen based on their observable color phenotype (88 fluorescent and 65 nonfluorescent) and the *egfp* gene sequenced. Of the 88 fluorescent variants sequenced, 42 different TNDs were identified and from the 65 nonfluorescent variants, 45 were unique TNDs; the total unique TNDs observed was 87. No wild-type EGFP was observed and no additional point mutations or frameshifts were observed in any of the sequenced variants. The distribution of the TNDs is shown in Figure 1A, with more detailed sequence information in Tables S1 and S2 available online. Observed mutations were distributed throughout the *egfp* gene, allowing thorough analysis of EGFP tolerance to single amino acid deletions. Due to the mechanism by which the library is constructed, the TND can span two codons and may give rise to a single amino acid deletion and an adjacent substitution mutation (Jones, 2005; Simm et al., 2007). Cross-codon TNDs can therefore introduce premature stop codons depending on the sequence surrounding the TND; this was observed for four variants and is likely to result in truncated nonfluorescent protein (Figure 1 and Table S2).

The position of the tolerated mutations in relation to the secondary and tertiary structure of EGFP is shown in Figures 1B and 1C. The majority of tolerated single amino acid deletions are found in loops connecting organized secondary structure (60%). The rest are equally distributed across helices (19%) and β strands (21%). The majority tolerated within β strands were found toward the strand termini, with the C-terminal ends of strands 7–11 particularly tolerant (Figure 1B). In relation to the tertiary structure, these sites translate to the two ends of the β barrel (Figure 1C).

This survey highlights the loops of EGFP as more tolerant to single amino acid deletions whereas β strands are the least tolerant. The proportion of EGFP comprising loops and β strands is 43% and 46%, respectively, which contrasts to the observed frequency of 60% and 21% of tolerated sites. In comparison, the observed frequency of tolerated positions in helical regions (19%) is ∼2 fold higher than helical contribution to the composition of EGFP secondary structure (11%). The N-terminal helix H1 and barrel capping helix H3 were particularly tolerant (Figure 1B). No tolerated sites were found in the core helical structure housing the chromophore. Of the 45 unique mutations giving rise to nonfluorescent variants, the majority (71%) are located in the middle of β strands, with 18% located in loops and 11% in helices (Figure S1 and Table S2). These included mutations that affected the three chromophore-forming residues, T65, Y66, and G67 (Table S2). Loops were not wholly tolerant to deletions but sensitive to the residue deleted. For example, removal of L137 in the long loop linking strands 6 and 7 was tolerated but deletion of residues D133 or G138 was not. Therefore, the residue removed in loops will dictate the structural rearrangements that occur rather than a general “whole loop” effect being observed.

**Figure 2:**
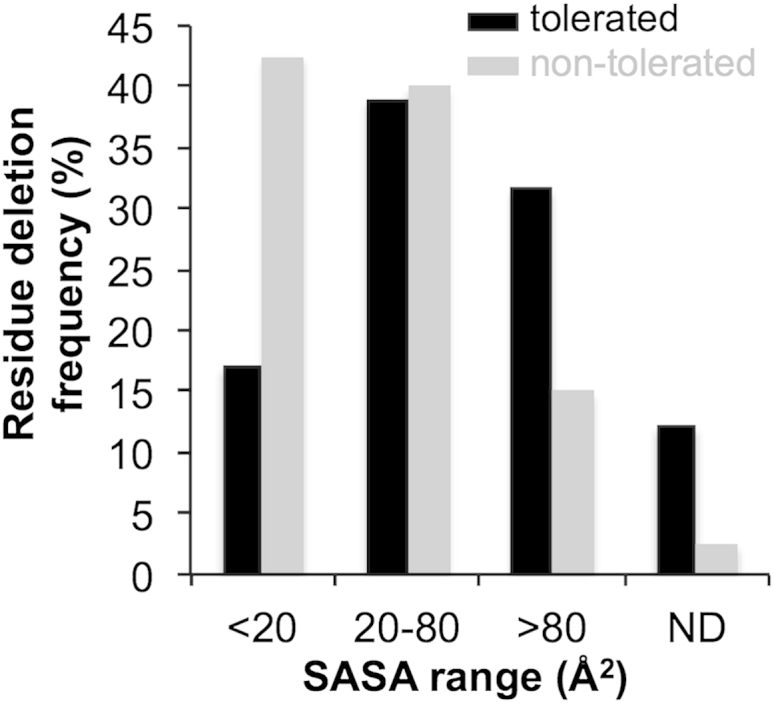
Relationship between EGFP and SASA Frequency of tolerance (black) and nontolerance (gray) of EGFP residues to deletion and their SASA. ND, not determined because their value could not be calculated because either the residue lies toward the N or C termini and are not part of the determined structure (PDB code: 4EUL; in the case of tolerated deletions) or form part of the chromophore (in the case of the nontolerated deletions).

The solvent-accessible surface area (SASA), an indicator of residue burial, of residues tolerant to deletion was relatively well distributed across the whole range with higher solvent exposed residues more tolerant (Figure 2 and Table S1). However, there was a trend toward lower SASA values for residues not tolerant to deletion (Figure 2 and Table S2). This stands to reason given that deletions like substitutions, of an amino acid buried in the core of a protein can be disruptive to protein structure and function. The relationship between tolerance to deletion and SASA has a link to the type of residue. Glycine and threonine residues are frequently observed to be tolerant deletions, both of which have relatively low inherent SASA. Larger residues such as leucine and tyrosine are less tolerant to deletion. Proline residues, including P89 involved in the sole *cis* peptide bond, appear to be tolerant to deletion; four of the five variants with a proline deleted still conferred a fluorescence phenotype on *E*. *coli* (Figure 1 and Tables S1 and S2).

### Identification and Fluorescence Properties of EGFP^G4Δ^

**Figure 3:**
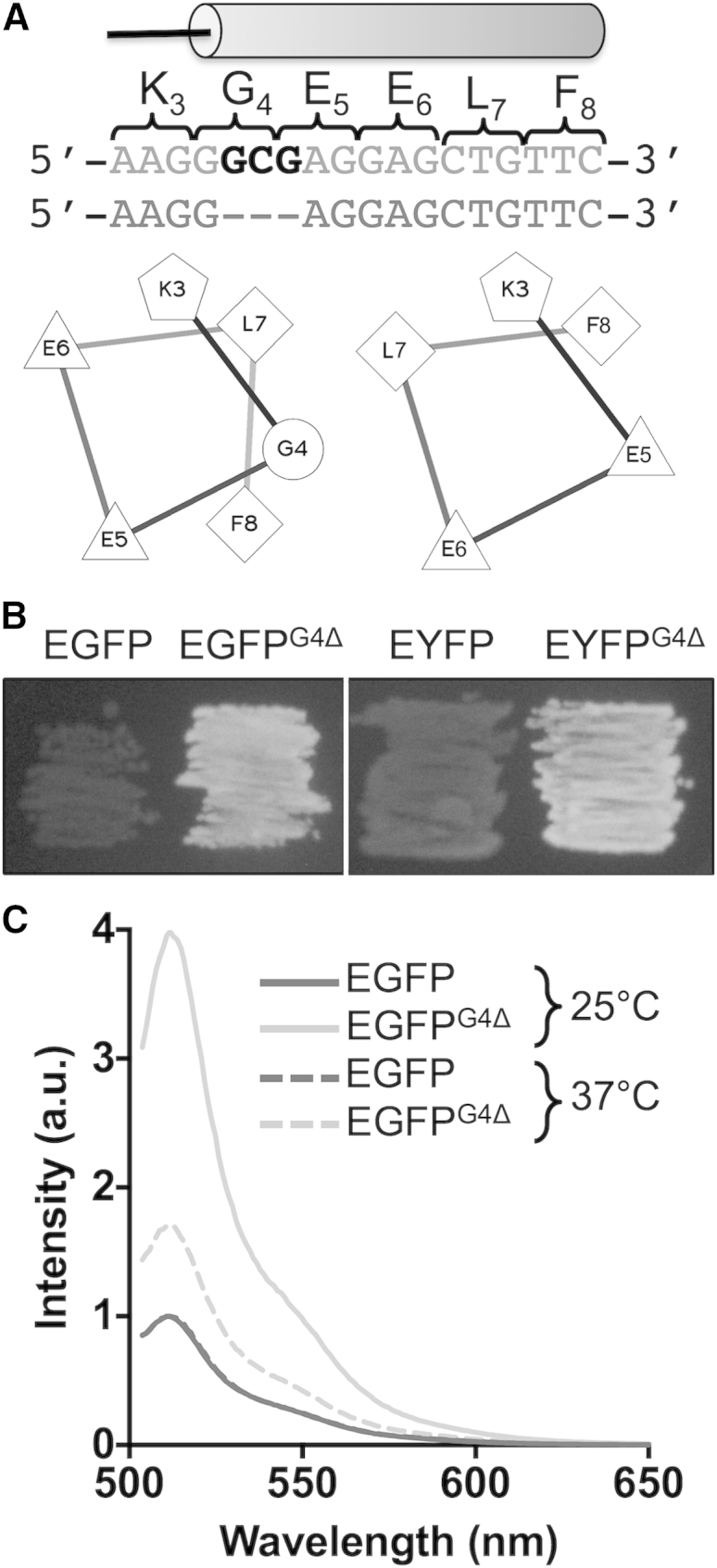
Fluorescence Properties of EGFP^G4Δ^ (A) The trinucleotide deletion giving rise to the G4Δ mutation and the potential helical register shift as represented by a helical wheel (hydrophobic, diamond; acidic, triangle; basic, pentagon). (B) Cellular fluorescence of the EGFP and EYFP G4Δ variants (color version available in Figure S2). (C) Whole cell fluorescence emission (excited at 488 nm) spectra for cultures grown at either 25°C (solid dashed lines) or 37°C (dashed lines). Cell cultures were standardized to an optical density 600 of 0.1 and the spectra normalized to EGFP fluorescence intensity.

Certain colonies appeared brighter than the general background level and sequencing revealed that the predominant mutation was G4Δ (where Δ refers to a deletion) resident in the N-terminal 3_10_ helix (H1; Figure 3A). Removal of G4 is likely to alter the registry (or relative side chain position) of the helix quite dramatically, as indicated by the helical wheel representation (Figure 3A). Cells expressing EGFP^G4Δ^ were visibly brighter than those expressing EGFP when grown in parallel on agar plates at 37°C (Figure 3B and Figure S2). The beneficial effects were found to be transferable as introduction of G4Δ to enhanced yellow fluorescent protein improved cellular fluorescence (Figure 3B). Analysis of whole cell fluorescence of cultures grown at 37°C (standardized to absorption at 600 nm of 0.1) induced for the same time revealed that those harboring EGFP^G4Δ^ were more fluorescent (∼2-fold) than EGFP; increased fluorescence intensity was even more pronounced (∼4-fold) for cultures grown at 25°C (Figure 3C). In vitro analysis of pure protein revealed that G4Δ did not affect EGFP fluorescence parameters (EGFP versus EGFP^G4Δ^) because the quantum yields (0.60 versus 0.59), mM extinction coefficients (55 versus 53), and fluorescence lifetimes (2.5 ns versus 2.6 ns) were very similar. This suggests that increased apparent brightness is the result of more efficient production of stable fluorescing protein in the cell rather than inherent changes to fluorescence.

### Folding Properties of EGFP^G4Δ^

**Figure 4:**
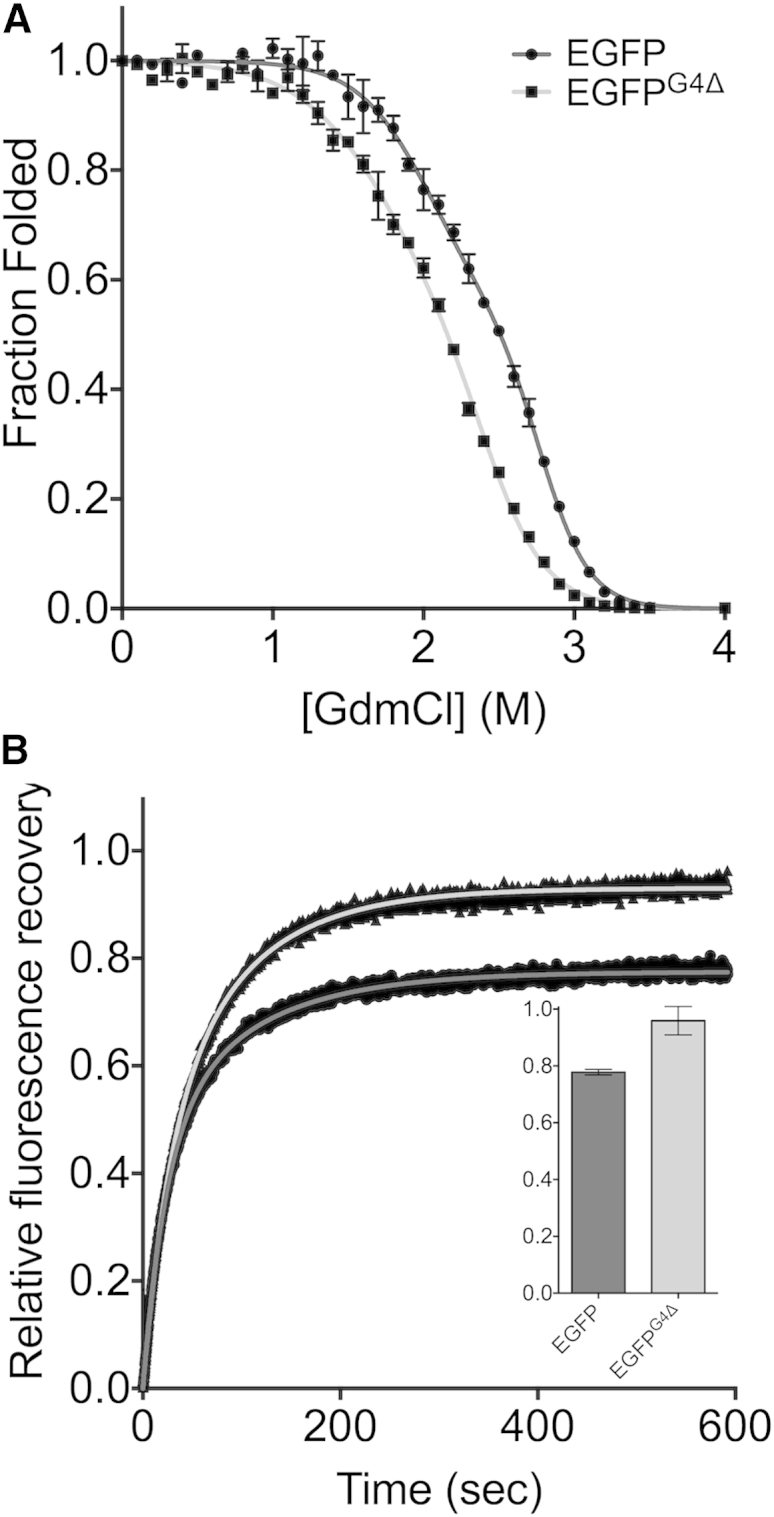
Folding Properties of EGFP^G4Δ^ (A) Equilibrium unfolding of EGFP (circles) and EGFP^G4Δ^ (squares). The curves were fit to a three-state model as outlined in the Experimental Procedures. Error bars represent SD of three replicates. (B) Refolding kinetics of EGFP (dark gray) and EGFP^G4Δ^ (light gray). The curves were fit to a double exponential as outlined in the Experimental Procedures and Supplemental Experimental Procedures. The final fluorescence recovery yield in refolding is shown in the inset graph.

Because there was little effect on intrinsic fluorescence, the influence of the G4Δ mutation on EGFP folding and stability was probed. It should be noted that EGFP is typical of similarly structured fluorescent proteins in that they are very resilient to chemical denaturation (Tsien, 1998) and take considerable time to reach equilibrium (>1 week; Figure S2; Hsu et al., 2009). Therefore, all reported equilibrium values are considered apparent even though after 250 hr incubation (time used in these studies) unfolding was approaching equilibrium based on the change in [GdmCl]_50%_ (Figure S3). The unfolding curves fit best to a three-state model (Figure 4A; see Figure S4 for fit to two-state model). The observed three-state unfolding suggests a folding intermediate is formed, as has been suggested for related GFPs (Hsu et al., 2009). The [GdmCl]_50%_^app^ for the native protein to intermediate transition (N-I) and intermediate to the denatured state transition (I-D) were similar, with EGFP^G4Δ^ being ∼0.3 M lower than EGFP at both transitions (Table 1). However, the dependencies of ΔG on [GdmCl] (or m values) for both transitions show a significant difference (Table 1). The m value for the N-I transition for EGFP is larger than that for EGFP^G4Δ^ by ∼0.65 kcal mol^−1^ M^−1^; this difference is more pronounced for the I-D transition with EGFP being ∼0.81 kcal mol^−1^ M^−1^ larger than EGFP^G4Δ^. The differences in m value thus have a major impact on the apparent stability (ΔG^H_2_^^O^_N-D_; Table 1), with EGFP^G4Δ^ unexpectedly destabilized by 5.19 kcal mol^−1^ compared to EGFP. However, ΔG^H_2_^^O^_N-D_ for EGFP^G4Δ^ is still relatively high (10.7 kcal mol^−1^) and far from being at the margin of stability. The melting temperature for thermal denaturation also remains high for EGFP^G4Δ^ (83°C) and is similar to that of EGFP (84°C; Figure S5).

While deleting G4 from EGFP appears to result in an overall decrease in stability, the m values provide insight into the structure of the intermediate and unfolded state. Because equilibrium unfolding m values are strongly correlated with a change in SASA, it is possible to estimate the change in surface area for the N-I and I-D transitions (calculated using http://www-clarke.ch.cam.ac.uk/BPPred.php; Geierhaas et al., 2007; Table 2). The calculated SASA for native EGFP is only slightly higher than that for EGFP^G4Δ^, differing by ∼550 Å^2^. For both transitions, the ΔSASA for EGFP^G4Δ^ is less than that for EGFP (ΔΔSASA_N-I_ = −1,620 Å^2^ and ΔΔSASA_I-D_ = −2,330 Å^2^). Because the calculated SASAs for native EGFP and EGFP^G4Δ^ are very similar, it appears that both the intermediate and the denatured forms of EGFP^G4Δ^ retain a greater degree of residue burial, implying a more compact structure compared to the same states for EGFP.

There were significant differences in the refolding kinetics of EGFP^G4Δ^ compared to EGFP. Importantly, up to 100% of the fluorescence signal observed before denaturation was recovered (Figure 4B), suggesting that a near full population of the protein molecules regained their native structure, comparable with the extensively mutated superfolder GFP (Andrews et al., 2007; Pédelacq et al., 2006). In comparison, 77% of EGFP fluorescence was recovered, consistent with previous observations for related “improved” GFP folding variants (Fukuda et al., 2000; Steiner et al., 2008). In both cases, refolding fit best to a double exponential with an initial fast phase (*k*_fast_) followed by a slow phase (*k*_slow_). The fast refolding phase for EGFP^G4Δ^ was marginally slower than EGFP but the slow refolding phase was marginally faster (Table 1). *Cis/trans* isomerization has been shown to be a rate-limiting step in protein folding (Wedemeyer et al., 2002) and is thought to be the reason for the slow refolding phase in GFP (Steiner et al., 2008). Therefore, the barrier to *cis*/*trans* isomerization appears to be slightly smaller for EGFP^G4Δ^.

### Structural Impact of G4Δ

The crystal structure of EGFP^G4Δ^ was determined to 1.5 Å resolution (Table 3 for statistics table) and compared to the recently determined high-resolution structure of EGFP (Arpino et al., 2012b; Royant and Noirclerc-Savoye, 2011). Size exclusion chromatography confirmed that like EGFP, EGFP^G4Δ^ is monomeric (Figure S6). The overall structures of EGFP and EGFP^G4Δ^ are very similar with backbone and all atom root-mean-square deviations of 0.6 Å and 1.2 Å, respectively (Figure S7), suggesting that deletion of G4 is having a subtle effect on structure, predominantly at the local level. E222, a critical residue in determining the protonated state of the chromophore phenol moiety (thus the spectral characteristics) (Tsien, 1998) and chromophore maturation (Sniegowski et al., 2005), exists in one of two distinct conformations in EGFP (Arpino et al., 2012b; Royant and Noirclerc-Savoye, 2011). However, the electron density for E222 in EGFP^G4Δ^ was best satisfied when modeled as a single distinct conformer equivalent to the major form in EGFP (Figure S8).

**Figure 5:**
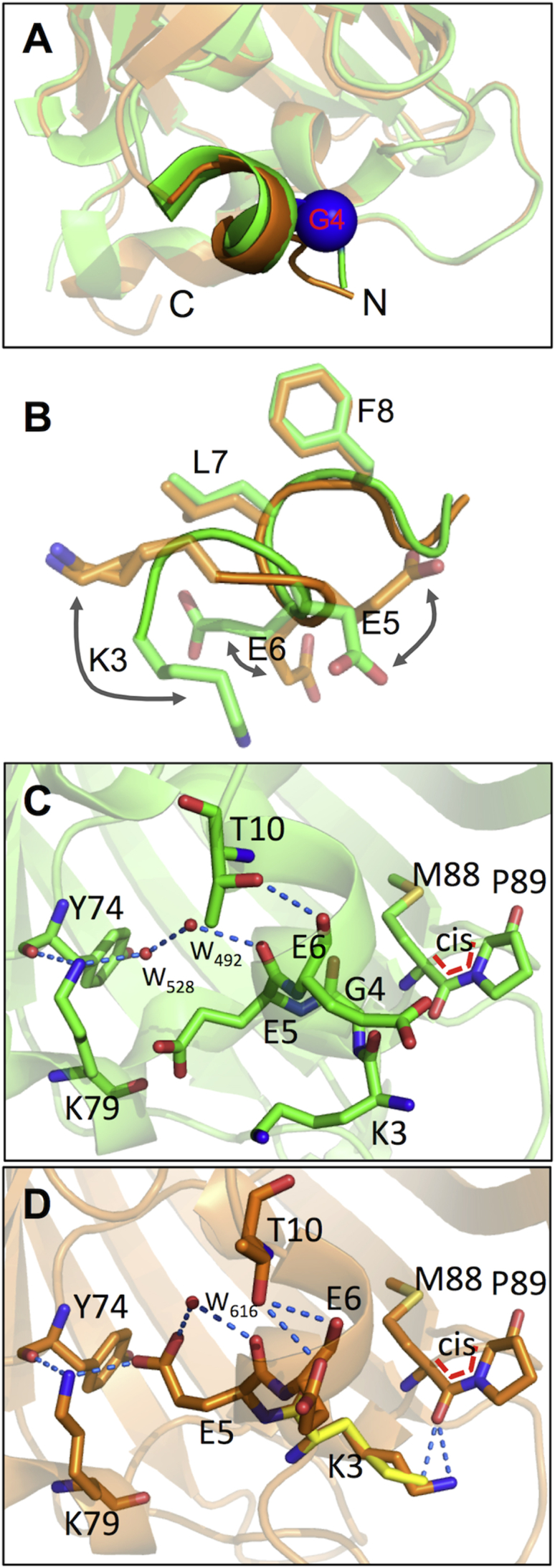
Structural Effects of the G4Δ Mutation on EGFP (A) Overlap of EGFP (green) and EGFP^G4Δ^ (orange) with the G4 residue in EGFP highlighted as a blue sphere. (B) The effect of G4 deletion on the side chain positioning of adjacent residues in the N-terminal 3_10_ helix. (C and D) Local structure of EGFP centered around G4 (C) and the rearrangements on deletion of G4 in EGFP^G4Δ^ (D) The alternative conformations for K3 are shown, with the second side chain conformer shown as yellow. The alternate conformations for K3 are highlighted in more detail in Figure S8.

In EGFP, G4 is located in the first organized structural element, a 3_10_ helix, which is relatively distant from the chromophore (Figure 5A and Figure S7). Deletion of G4 results in a significant local rearrangement of residues in the 3_10_ helix and the helix itself (Figure 5B). K3 rotates by ∼120° around the axis of the 3_10_ helix to reside at the position previously occupied by G4. Modeling the K3 side chain to two conformations that differ slightly (root-mean-square deviation 0.66 Å for A versus B) best satisfied the electron density during structure refinement (Figure S9). The side chain positioning of E5 and E6 are also significantly perturbed, but register is restored from L7 and F8 onward (Figure 5B).

The shift of K3 positioning in EGFP^G4Δ^ appears to have two main effects on the local structure. First, rotation brings the K3 side chain into the vicinity of the *cis* peptide bond between M88 and P89 with the N^Z^ group being within hydrogen bonding distance of the backbone carbonyl O of M88 (Figures 4D and 5C). This in turn could have implications in stabilization of the *cis* peptide bond. Second, the shift in the 3_10_ helix to accommodate the K3 side chain repositions E5 and generates a new polar network (Figures 5C and 5D). The carboxylate group of E5 is now within electrostatic bonding distance (2.7 Å) of the N^Z^ amine group of K79 in the adjacent 3_10_ helix, which in turn is within hydrogen bond distance of the backbone carbonyl group of Y74 (Figure 5D). This new polar bond network absent from EGFP now links different secondary structure elements in EGFP^G4Δ^.

There are also changes to the arrangement of structured water molecules (Figures 5C and 5D). E5 side chain rearrangement appears to result in the displacement of a water molecule normally observed in EGFP (W_528_ in Figure 5C). The position of a second water molecule (W_492_ in EGFP and W_616_ in EGFP^G4Δ^) is similar between the two but is now capable of forming a hydrogen bond with the E5 side chain in EGFP^G4Δ^.

## Discussion

Predicting the effects of backbone mutations such as single amino acid deletions and the implications in terms of protein structure is currently very difficult and generally avoided as part of the protein design process. This is because not only do deletion mutations alter side-chain placement of adjacent residues, but also locally confine structure. Using directed-evolution approaches, a survey of tolerated deletion mutations can be conducted, which in turn can unearth mutations not obvious on initial inspection that enhance certain properties of a protein in unpredicted ways. InDel events involving GFP, whether they are small alterations such as those here or more drastic events such as insertions of whole protein domains (Arpino et al., 2012a; Baird et al., 1999; Biondi et al., 1998; Doi and Yanagawa, 1999) can lead to new and/or improved functionality so should not be ignored as useful routes to protein engineering. This in turn provides us with mechanistic insights concerning how InDel mutations exert their effect on protein structure during the natural evolutionary process and allow feedback to the protein design process.

Fluorescent proteins represent an important and current target for protein engineering (Pakhomov and Martynov, 2008; Tsien, 1998). Only limited deletion mutations targeted at the termini (Dopf and Horiagon, 1996; Flores-Ramírez et al., 2007; Li et al., 1997) and selected loops (Flores-Ramírez et al., 2007; Li et al., 1997) has been performed on proteins related to GFP. This study is more comprehensive in terms of assessing tolerance and impact throughout the protein. Of the 87 unique deletion events observed, 42 (48%) were tolerated. This is in contrast to previous amino acid deletion studies, which suggest GFP to be largely intolerant to amino acid deletions (Flores-Ramírez et al., 2007; Li et al., 1997). Indeed, such a high frequency of tolerance suggests protein structure is suitably plastic to incorporate backbone alternations without complete loss of stability and function. Recent bioinformatics studies of proteomes suggests that InDel mutations, especially deletion mutations, are major instigators of leaps in the fitness landscape of a protein but largely require local substitution mutations to elicit an effect (Leushkin et al., 2012; Tóth-Petróczy and Tawfik, 2013); this does not appear to always be the case in the more directed approach taken here where single deletions can not only be tolerated, but also be beneficial when incorporated alone (vide infra).

Analysis of the tolerated and nontolerated amino acid deletion positions by mapping to the secondary structure topology and tertiary structure of EGFP (Figure 1 and Figure S1) showed a clear divide between regions tolerant and nontolerant to deletion mutagenesis. Our results here agree to an extent with the dogma that deletion mutations are better tolerated in loops rather than ordered secondary structure (Pascarella and Argos, 1992). However, helical segments appear more tolerant to deletion than strands (Figures 1B and 1C). Deletion within a strand may cause registry shifts and given EGFP fluorescence is reliant on its tertiary structure, will have obvious detrimental effect on function, the primary screening property. Termini of strands appear to be more tolerant to residue removal, with 21% of tolerated deletions in these regions (Figure 1B). This is in line with previous observations with TEM β-lactamase (Simm et al., 2007). As observed previously with TEM β-lactamase (Simm et al., 2007), helical structures appear more resilient to deletions (Figures 1B and 1C). In EGFP, H1 and H3 were particularly tolerant. Whereas the general tolerance of H1 to deletion may not be entirely surprising given that removal of the first five amino acids can be tolerated to a degree (Li et al., 1997), at first glance the beneficial effects are unexpected: one of the enhanced variants, EGFP^G4Δ^, has residue from H1 removed (vide infra). Thus, deletions of residues within helices may not be as disruptive as within strands, which may be a consequence of the “stand-alone” nature of helices compared to a strand that forms one element of a β sheet system. This is especially pertinent in the case of EGFP whereby strands form a critical structural feature of the protein (the β barrel) with the two faces of the strands differing markedly in their chemical composition. Structural analysis revealed that helices soon regain residue register (Figure 5B). The relative surface burial of a residue may not be such a critical factor in defining tolerance because deletion of residues with a wide range of SASA values was allowed. However, residues with low solvent exposure have a higher propensity to be nontolerant to deletion (Figure 2). For example, the buried central core helix was not tolerant but it is unknown whether these deletions proved disruptive to the β barrel structure as a whole or to chromophore maturation (and thus fluorescence) because two deletion mutations removed chromophore-forming residues (Figure S1 and Table S2).

Whereas loops are the most tolerant structure to deletions in terms of EGFP, many of these deletion mutations where observed in short loops (five residues or less) that could be considered as turns between organized secondary structure elements. Most notable were short loops connecting S2-S3, S3-H2, S7-S8, and S8-S9 (Figure 1B). Shortening of these already constrained loops could be considered deleterious, but this does not appear to be the case. This is backed up to an extent by the few observed deletion mutations in short loops of nonfluorescent variants (Figure S1), suggesting they are more tolerant than would be expected. These short turn loops in EGFP are also tolerant to larger InDel events such as domain insertion to generate new protein scaffolds with coupled activities (Arpino et al., 2012a).

The context of the deletion appears to be a more important determinant than the secondary structure it occupies. This is illustrated by H1, a 3_10_ helix largely tolerant to deletions. However, only deletion of G4 results in the local structural rearrangement resulting in improved cellular fluorescence through potential stabilization of the *cis* M88-P89 peptide bond (Figure 5). Deletion of the adjacent E5 and E6 residues has little effect on fluorescence phenotype (Table S1) and loss of K3 with the G4S mutation renders the protein nonfluorescent (Table S2). Even replacement of K3 with N removes the beneficial properties of G4Δ (Table S1 and Figure S10), probably by eliminating the favorable interaction the K3 amine group makes with the *cis* M88-P89 peptide bond (vide infra). Key to the beneficial mutational mechanism is the side-chain “flipping” or registry shift (Figure 5B), an event considered to be deleterious, in making new long-range interactions.

Deletion of G4 promotes increased production of functional protein in the cell. Rather than affecting brightness or apparent thermodynamic stability of EGFP, the influence of G4Δ is likely to be manifested through changes in/optimization of the folding process and avoiding potential off-pathway aggregation. Apparent thermodynamic stability of EGFP^G4Δ^ is curiously lower than that of EGFP (Figure 4A and Table 1), but functional recovery after denaturation is higher (Figure 4B). Unlike many stabilized GFP derived variants containing multiple substitutions (e.g., GFPmut2, Cannone et al., 2005; and cycle 3 GFP, Fukuda et al., 2000 variants), up to 100% of EGFP^G4Δ^ refolds to a functional state after denaturation (Figure 4B). Given the importance of the folding process to proteins in general, deletion mutations need not be considered harmful and thus be used generally as a mechanism to improve proteins. This has been demonstrated to a degree here by transplanting the G4Δ mutation to EYFP and increasing observed cellular fluorescence (Figure 3B).

The unfolding and refolding properties for EGFP presented here are in good agreement with previous work that uses the *p*-hydroxybenzylidene-imidazolinone (HBI) chromophore as a probe to monitor (un)folding (Hsu et al., 2009; Steiner et al., 2008; Stepanenko et al., 2004). Both EGFP and EGFP^G4Δ^ equilibrium unfolding fitted to a three-state model, suggesting that folding of the mature protein occurs via an intermediate, which has been observed previously for other related GFPs (Andrews et al., 2008, 2009; Hsu et al., 2009). Although EGFP^G4Δ^ is less stable by an apparent ΔΔG_N-D_ of 5.1 kcal/mol due predominantly to the change in m value for both transitions (native to intermediate and intermediate to denatured), the m values themselves suggest a change in the folding process itself especially regarding the degree of accessible surface area (Myers et al., 1995). The predicted changes in SASA for EGFP^G4Δ^ on unfolding are lower than those for EGFP, suggesting that the deletion variant retains a more compact structure in the intermediate and denatured forms. The intermediate state is already considered highly structured in EGFP (Table 1 and Table S3) and in other engineered GFPs (Andrews et al., 2007, 2008, 2009; Huang et al., 2007; Xie and Zhou, 2008), with deletion of G4 potentially increasing it. The apparent increased residue burial/structure in the intermediate and denatured state on introduction of G4Δ may be a function of the long-range polar interactions observed for mature, native EGFP^G4Δ^ (Figure 5D). The putative hydrogen bond via the K3 amine with the carbonyl group of the M88-P89 peptide bond may stabilize the sole *cis* proline peptide bond and promote conversion to the native state. Stabilization of the *cis* proline peptide bond has implications in terms of backbone trajectory flux during folding that assists transition to the native state. *Cis-trans* isomerization around the M88-P89 peptide bond is known to be important to the folding process (Enoki et al., 2004) and is considered to be the rate-limiting step in folding (Hsu et al., 2009). *Cis-trans* isomerization around a X-Pro peptide bond is in general one of the rate-determining steps in protein folding and can deviate a protein away from its native folding trajectory to a kinetically trapped aggregative state if the incorrect forms persist (Steiner et al., 2008; Stepanenko et al., 2004). If stabilization of the M88-P89 *cis* peptide bond is perpetuated in the intermediate (and even to a degree in the denatured) state, it may explain the decreased SASA inferred from the m values for EGFP^G4Δ^ due to a higher degree of residual and/or transient structure. Both G4Δ and the M88-P89 *cis* proline are located in regions that contribute toward the structured element of the equilibrium folding intermediate (Huang et al., 2007; Reddy et al., 2012).

The G4Δ mutation only slightly sped up the slow phase of EGFP folding, but must be put in context of an increase in folding efficiency (Figure 3B). Recent folding simulations of a related GFP, citrine, revealed that misplacement of a loop connecting strands S9 and S10 resulted in the protein becoming stuck in a misfolded kinetic trap (Reddy et al., 2012). This loop lies adjacent to the *cis* M88-P89 peptide bond, which is in turn stabilized through interactions with the repositioned K3 on G4 deletion. Furthermore, residues in sfGFP forming the lid of the β barrel (including K3 and G4), an element that plays a key role in driving the intermediate to the fully folded fluorescent protein, display conformation heterogeneity in the native state (Andrews et al., 2009). Thus, the role of G4Δ may lie in optimization of the folding efficiency and mechanism.

The shift in register of H1 on deletion of G4 has additional effects in conjunction with repositioning K3 through the formation of an extended polar interaction network (Figure 5). Accompanying the changes involving K3 is the repositioning of E5 resulting in direct interaction with K79 on an adjacent secondary structure element. The generation of such networks has been observed before in fast and efficient folding versions of GFP (Pédelacq et al., 2006).

The one curious and counterintuitive observation is the significant decrease in apparent thermodynamic stability, especially given the generally stabilizing interactions formed on deleting G4. The increased structure in the intermediate and denatured states relative to the native state as implied by the m values may have an impact on the apparent thermodynamic stability of mature EGFP^G4Δ^ through changes in absolute free energy levels. Thus, the role of G4Δ may lie in optimization of the refolding efficiency and mechanism at the expense of an improved ΔG_N-D_. However, it cannot be discounted that the HBI chromophore used to probe folding is more sensitive to changes in EGFP^G4Δ^ structure, which may be affecting values related to stability such as [GdmCl]_50%_. Although there is generally good correlation between different probes to monitor GFP unfolding, there can be slight discrepancies in the [GdmCl]_50%_ value (Huang et al., 2007). The G4Δ mutation may have a greater impact on the de novo folding of the nascent GFP prior to chromophore formation than the refolding/unfolding of the mature protein. Folding of GFP is known to be dependent on the chromophore and has been suggested as the cause for the “hysteresis” folding phenomenon observed for mature GFP (Hsu et al., 2009) due to a “dual basin” folding landscape comprising a native-like intermediate and the “locked” native, fluorescent state (Andrews et al., 2008). Proline isomerization, especially with regards to P89, together with formation of the β barrel lid (vide supra) is thought to be an important contributor to the barrier between the two states. While de novo folding has not been explored here, it may explain why G4Δ results in higher cellular fluorescence in situ (Figure 3). Chromophore maturation occurs after folding and depends on formation of a correctly folded protein (Reid and Flynn, 1997). Furthermore, the N-terminal region is thought to facilitate the folding process of the nascent polypeptide on release from the ribosome (Uemura et al., 2008). Therefore, G4Δ may be having a more significant impact on folding immediately after release from the ribosome in the complex mixture of the cell where off-pathway aggregative folding events are more likely.

Contrary to current dogma, deletion of a single amino acid is generally well tolerated throughout a protein, including helical elements, and can be beneficial as exemplified by the G4Δ mutation. The thought that amino acid deletions hinder protein folding conflicts with observations here as the basis for improved cellular fluorescence imparted by EGFP^G4Δ^ is thought to be due to changes in the folding process. The residue repositioning through a helical registry shift is critical to generating a new interaction network and is unlikely to have risen through substitution mutations alone, highlighting the ability of deletion mutations to sample structural space not accessible through exchanging only side chains. Indeed, the influence of deletion mutations can go beyond structural stability and also influence functional properties (Fujii et al., 2006; Simm et al., 2007). The identification of G4, which is so close to the start of the structured region of EGFP (Arpino et al., 2012b) highlights the sometimes nonintuitive nature of useful deletion mutations and bestows the benefits of a directed-evolution approach. Together with work presented here, the recent idea of InDel mutations instigating major leaps in the protein fitness landscape during evolution, with compensating (or enabling) substitution mutations (mostly local to the InDel event) helping to improve overall fitness (Leushkin et al., 2012; Tóth-Petróczy and Tawfik, 2013), potentially provides a template for future protein engineering strategies.

## Experimental Procedures

### TND Library Construction

Insertion of the engineered transposon MuDel (Jones, 2005) into the *egfp* gene encoding EGFP residing within the pNOM-XP3 plasmid was performed using an in vitro transposition and selection procedure described elsewhere (Baldwin et al., 2009) to generate the library *egfp*Δ^2504^. This is described in more detail in the Supplemental Experimental Procedures.

### Protein Production and Purification

The production and subsequent purification of EGFP and EGFP^G4Δ^ was performed essentially as described elsewhere (Arpino et al., 2012b). A detailed procedure is provided in the Supplemental Experimental Procedures. The production of EGFP and EGFP^G4Δ^ for whole cell fluorescence analysis was performed as follows. Luria-Bertani (LB) broth (20 ml) supplemented with 100 μg/ml ampicillin and 1 mM isopropyl-beta-D-thiogalactopyranoside (IPTG) was inoculated with a single *E*. *coli* BL21-Gold (DE3) colony containing a relevant plasmid (pNOM-XP3 containing the *egfp* gene or *egfp*^*G4*Δ^ genes) and incubated overnight at either 25°C or 37°C. The production of EGFP, EGFP^G4Δ^, EYFP, and EYFP^G4Δ^ in colonies streaked out on LB agar plates was performed as follows. A single BL21-Gold (DE3) colony containing the relevant plasmid (pNOM-XP3 containing the *egfp, egfp*^*G4*Δ^, *eyfp*, or *eyfp*^*G4*Δ^ genes) was resuspended in LB broth (200 μl) supplemented with 100 μg/ml ampicillin and incubated at 37°C shaking (200 revolutions per minute) for 2 hr. The cultures were streaked out onto LB agar plates supplemented with 100 μg/ml ampicillin and 150 μM IPTG. The plates were incubated overnight at 37°C and depicted with a transilluminator.

### Fluorescence Spectroscopy

Excitation and emission spectra were measured using a Cary Eclipse fluorescence spectrophotometer (Varian) in a cuvette of dimensions 5 × 5 mm with a 10 nm excitation and emission band pass at a scan rate of 600 nm/min. Excitation scans were measured by monitoring emission at 511 nm and emission was measured after excitation at 488 nm. Whole cell fluorescence spectroscopy was performed on *E*. *coli* BL21-Gold (DE3) cell cultures after expression of EGFP and EGFP^G4Δ^ at either 25°C or 37°C. Expression cultures were harvested by centrifugation (1,500 × *g* for 10 min) and all supernatant removed and discarded. The cell pellet was resuspended in 50 mM Tris-HCl, pH 8.0 at 25°C, 150 mM NaCl, and 10% (v/v) glycerol (TNG Buffer) to an optical density 600 of 0.1 in a 1 cm path length cuvette. The resuspended cells were transferred to a cuvette with 5 × 5 mm dimensions and excitation and emission spectra measured as described above. Fluorescence measurements using purified protein samples were performed in 50 mM Tris-HCl, pH 8.0 at 25°C, and 150 mM NaCl. The calculation of quantum yield and fluorescence lifetimes were performed as described elsewhere (Arpino et al., 2012a, 2012b).

### Guanidine Hydrochloride Equilibrium Unfolding

Purified protein (1 μM) was prepared in TNG Buffer and guanidine hydrochloride (0–6 M GdmCl) and incubated for up to 250 hr at 37°C. Protein unfolding was monitored by fluorescence at 520 nm after excitation at 480 nm using a FLUOstar Omega microplate reader (BMG Labtech). To estimate the apparent [GdmCl] at which 50% of the protein is folded and 50% of the protein is unfolded, samples were incubated in 96-well plates at 37°C and measured after 2.5, 5, 22, 48, 120, and 250 hr.

Equilibrium unfolding data measured from samples incubated at 37°C in Eppendorf tubes for 250 hr were fit to a three-state model (Equation 1) formulated using the following equations:

$$K_{N-I}={exp}^{\left(\frac{m_{N-I}[D\}-\Delta G_{N-I}}{RT}\right)},\;K_{I-D}=exp^{\left(\frac{m_{I-D}[D\}-\Delta G_{I-D}}{RT}\right)}$$

$$Fr_{N}=\frac{1}{1+K_{N-I}+K_{N-I}K_{I-D}},\;Fr_{I}=\frac{K_{N-I}}{1+K_{N-I}+K_{N-I}K_{I-D}},\;Fr_{D}=\frac{K_{N-I}K_{I-D}}{1+K_{N-I}+K_{N-I}K_{I-D}}$$

(Equation 1) $$F=Y_{N}+Fr_{I}\left(Y_{I}-Y_{N}\right)+Fr_{D}\left(Y_{D}-Y_{N}\right),$$

where ΔG_N-I_ is the difference in Gibbs’ free energy between native and intermediate states and ΔG_I-D_ is the difference between intermediate and denatured states. *m*_N-I_ is a constant that describes the dependence of ΔG on denaturant concentration, [D], between the native and intermediate states, whereas *m*_I-D_ is the same for the intermediate to denatured states. *Fr*_N_, *Fr*_I_, and *Fr*_D_ are fractions of the partition function in a three-energy-state system, and the plot of fractional populations of different states against denaturant concentration can be generated from these equations.

### Protein Refolding Kinetics

Protein samples were unfolded by dilution (1/100) to a final concentration of 1 μM in 6.4 M GdmCl and refolded by rapid dilution (1/10) into fresh TNG Buffer supplemented with 1 mM dithiothreitol so that the final protein concentration was 100 nM and GdmCl was 0.64 M. In both cases, fluorescence was monitored for 20 min at 510 nm after excitation at 488 nm in a 5 × 5 mm dimension cuvette with an excitation and emission band pass of 2.5 and 5 nm, respectively. Unfolding data were fit with a single exponential decay (Equation 2) and refolding data fit with a double exponential (Equation 3):

(Equation 2) $$Y=\left(Y_{0}-P\right)\times e^{-kt}+P$$

(Equation 3) $$Y=Y_{0}+\left(F_{1}\times \left(1-e^{-k_{fast}t}\right)\right)+\left(F_{2}\times \left(1-e^{-k_{slow}t}\right)\right),$$

where Y_0_ is the Y value when t = 0; P is the Y value at infinite time; F_1_ is a proportional value for the first rate constant, *k*_*fast*_; and F_2_ is the proportional value for the second rate constant, *k*_*slow*_.

### Protein Crystallization and Structure Determination

Purified EGFP^G4Δ^ (15 mg/ml in 50 mM Tris-HCl, pH 8.0, and 150 mM NaCl) was screened for crystal formation by the sitting drop vapor diffusion method with incubation at 18°C. Drops were set up with equal volumes of protein and precipitant solutions (0.5 μl each). Crystals of EGFP^G4Δ^ were obtained from 0.1 M HEPES, pH 7.0, 0.01 M ZnCl_2_, and 20% (w/v) PEG 6000. Data were collected on beamline I03 at the Diamond Light Source, Harwell, UK. Usable diffraction was recorded up to a resolution of 1.58 Å. Data were reduced with the XIA2 package (Winter, 2009), space group assignment was done by POINTLESS (Evans, 2006), and scaling and merging were completed with SCALA (Evans, 2006) and TRUNCATE (CCP4, 1994). Initial molecular replacement for the EGFP^G4Δ^ variant structure was performed using a previously determined EGFP structure (Protein Data Bank [PDB] entry 4EUL; Arpino et al., 2012a) as the search model, using PHASER (McCoy et al., 2007). The structure for EGFP^G4Δ^ was adjusted manually using COOT (Emsley and Cowtan, 2004) and refinement of the completed molecule was carried out using REFMAC (Murshudov et al., 1997). Protein atoms were refined isotropically and anisotropically. All nonprotein atoms were refined isotropically. The above routines were used within the CCP4 package (CCP4, 1994; http://www.ccp4.ac.uk). Graphical representations were made with PyMOL Molecular Graphics System (Schrödinger).

## Figures and Tables

**Table 1:** Equilibrium Unfolding, Unfolding, and Refolding Kinetic Parameters

Variant	D_N-I_ (M)^a^	*m*_N-I_ (kcal mol^−1^ M^−1^)^b^	D_I-D_ (M)^c^	*m*_I-D_ (kcal mol^−1^ M^−1^)^b^	ΔG_*N-I*_	ΔG_*I-D*_	ΔG_*N-D*_	*k*_U_ (min^−1^)^e^	*k*_fas_ (10^−2^ s^−1^)^f^	*k*_slow_ (10^−2^ s^−1^)^f^
					(kcal mol^−1^)^d^					
EGFP	2.04	2.55 ± 0.23	2.79	3.73 ± 0.27	5.18	10.37	15.55	2.09 ± 0.00	4.32 ± 0.08	1.00 ± 0.02
EGFP^G4Δ^	1.76	1.96 ± 0.1	2.43	2.89 ± 0.14	3.46	7.01	10.47	2.15 ± 0.01	3.78 ± 0.25	1.15 ± 0.05

a Concentration of GdmCl at which 50% of the protein sample is in the native and intermediate state.

b Measure of dependence of ΔG on denaturant concentration, m value. Error bars represent 1 SD from three replicates.

c Concentration of GdmCl at which 50% of the protein sample is in the intermediate and denatured state.

d Change in free energy for native to intermediate (N-I), intermediate to denatured (I-D), and native to denatured (N-D) transitions.

e Rate constant from single exponential fit of unfolding progress curves (data not shown). Error bars represent 1 SD from three replicates.

f Rate constants from two exponential fits of refolding progress curves (Figure 2B). Error bars represent 1 SD from three replicates.

**Table 2:** Solvent-Accessible Surface Area Changes on Unfolding

Protein	Native SASA (Å^2^)^a^	Fully Unfolded SASA (Å^2^)^a^	ΔSASA_N-I_ (Å^2^)^b^	ΔSASA_I-D_ (Å^2^)^b^	ΔSASA_N-D_ (Å^2^)^b^
EGFP	9,919	31,849	7,050 ± 340	10,320 ± 440	17,370 ± 390
EGFP^G4Δ^	9,366	31,953	5,430 ± 300	7,990 ± 370	13,420 ± 350

a Calculated using the calc-surface program (http://helixweb.nih.gov/structbio/basic.html). Only residues K3–L231 were considered for SASA calculations. Unfolded state refers to fully unfolded peptide.

b Calculated with the determined m values using BPPred (http://www-clarke.ch.cam.ac.uk/BPPred.php; Geierhaas et al., 2007).

**Table 3:** Crystallographic Statistics

Data Reduction Statistics	EGFP^G4Δ^
Beamline	I03
Wavelength (Å)	0.97630
Space group	C121
a, b, c (Å)	91.9, 66.7, 45.3, β = 108.76°
Resolution range (Å)	43.49–1.58
Total reflections measured	125,853
Unique reflections	34,564
Completeness (%) (last shell)	97.5 (98.2)
I/σ (last shell)	8.9 (2.1)
R(merge)^a^ (%) (last shell)	8.4 (53.8)
B(iso) from Wilson (Å^2^)	12.1

**Refinement Statistics**	

Protein atoms excluding H	1,987
Solvent molecules	270
R-factor^b^ (%)	17.3
R-free^c^ (%)	21.10
Rmsd bond lengths (Å)	0.024

**Ramachandran Plot Statistics**	

Rmsd angles (°)	2.3
Core region (%)	96.7
Allowed region (%)	3.3
Additionally allowed region (%)	0
Disallowed region (%)	0
PDB code	4KA9

Rmsd, root-mean-square deviation.

a $R_{merge}=\sum_{h}\sum_{j}\left(I_{hj}-\langle I_{j}\rangle \right)/\sum_{h}I_{hj}.$

b $R_{factor}=\left(\sum_{h}\left|F_{h,obs}-F_{h,calc}\right|\right)/\left(\sum_{h}F_{h,obs}\right).$

c *R*_*free*_ is calculated from a set of 5% randomly selected reflections that were excluded from refinement.

## Accession Numbers

The PDB accession number for the EGFP^G4Δ^ structure is 4KA9.

## Supplemental Information

Document S1. Supplemental Experimental Procedures, Figures S1–S10, and Tables S1 and S2

Document S2. Article plus Supplemental Information
