# Supplementary material for: Random Single Amino Acid Deletion Sampling Unveils Structural Tolerance and the Benefits of Helical Registry Shift on GFP Folding and Structure
Source: Structure. 2014 Jun 10;22(6):889–98. doi: 10.1016/j.str.2014.03.014 (PMC4058518; doi:10.1016/j.str.2014.03.014)
Supplement: Document S1. Supplemental Experimental Procedures, Figures S1–S10, and Tables S1 and S2 [file mmc1.pdf]

Structure, Volume *22*

Supplemental Information

Random Single Amino Acid Deletion Sampling Unveils Structural Tolerance and the Benefits of Helical Registry Shift on GFP Folding and Structure

James A. J. Arpino, Samuel C. Reddington, Lisa M. Halliwell, Pierre J. Rizkallah, and D. Dafydd Jones

Random single amino acid deletion sampling unveils structural tolerance and the benefits of helical registry shift on GFP folding and structure.

James A. J. Arpino[1], Sam C. Reddington[1], Lisa M. Halliwell[1], Pierre J. Rizkallah[2] & D. Dafydd Jones.[1]

Supporting Information.

Supporting Methods.

EGFP TND library construction.

Insertion of the engineered transposon MuDel into the *egfp* gene encoding enhanced green fluorescent protein (EGFP) residing within the pNOM-XP3 plasmid was performed using an *in vitro* transposition and selection procedure described previously (Baldwin et al., 2009) to generate the library *egfp*Δ^{2504}. *Mly*I restriction digestion was performed on *egfp*Δ^{2504} DNA (3 μg) to remove MuDel from the pooled plasmid library and analysed by 1.0% (w/v) agarose gel electrophoresis. The linear library DNA was purified from the agarose gel using a QIAquick® gel purification kit (QIAGEN). The purified linear library DNA (50 ng) was recircularised by intramolecular ligation with Quick T4 DNA ligase and the reaction cleaned up with a MinElute reaction cleanup kit (QIAGEN). The ligation reaction mixture (1 μl) was used to transform electrocompetent *E. coli* BL21-Gold (DE3) cells. The transformed cells were grown on LB agar plates supplemented with 100 μg/ml ampicillin and 150 μM IPTG and incubated at 37°C overnight then stored at 4°C. Colonies presenting a green colour phenotype upon illumination on a UV transilluminator and colonies with no colour phenotype were selected for a colony PCR screen with primers pEXP-F and DDJ013. The PCR products produced (2 μl) were analysed by agarose gel electrophoresis and the rest (23 μl) purified using a QIAquick PCR purification kit (QIAGEN) for DNA sequence analysis, to identify the nature of the triplet nucleotide deletions.

Protein production and purification

The production and subsequent purification of EGFP and EGFP$^{G4\Delta}$ was performed as follows. LB Broth (15 ml) supplemented with 100 μg/ml ampicillin was inoculated with a single *E. coli* BL21-Gold (DE3) colony containing a relevant plasmid (pNOM-XP3 (Baldwin et al., 2009) containing the *egfp* or *egfp*$^{G4\Delta}$ gene) to generate a starter culture and incubated overnight at 37°C. A 1/200 dilution of the starter culture was used to inoculate 1l of LB broth supplemented with 100 μg/ml ampicillin and grown at 37 °C until an O.D.600 of 0.4-0.8 was achieved. Protein expression was induced by the addition of 1 mM IPTG and incubated for 24 hrs at 37 °C. The 1l culture was harvested by centrifugation (3000 x g for 20 mins) and the pellet resuspended in 25 ml 50 mM Tris-HCl, pH 8.0 (Buffer A) and supplemented with 1 mM phenylmethanesulfonylfluoride (PMSF) and 1 mM ethyldiaminetetraacetic acid (EDTA). The cells were lysed by French press using a chilled pressure cell. The lysate was then centrifuged (20000 rpm in a Beckman JA20 rotor for 30 mins) to pellet any cell debris and the supernatant was decanted and stored at 4°C. The cell lysate was subjected to fractionation with ammonium sulphate precipitation. An initial ammonium sulphate concentration of 45% (w/v) was used to precipitate unwanted proteins from solution. After clearance of unwanted precipitate by centrifugation (20000 rpm in a Beckman JA20 rotor for 40 mins) further addition of ammonium sulphate to a final concentration of 75% (w/v) was carried out to precipitate EGFP or EGFP$^{G4\Delta}$. The precipitate was resuspended in 5 ml Buffer A. The sample was buffer exchanged into fresh Buffer A by dialysis in a 10000 MWCO membrane to

remove any remaining ammonium sulphate. A precipitate formed during dialysis and was removed by centrifugation at 10,000 rpm in a Beckman JA-20 rotor for 20 min. The supernatant was applied to a Resource Q (GE Healthcare) anion exchange column (5 ml bed volume, flow rate 2 ml/min) equilibrated with Buffer A. Target proteins were eluted using a gradient from 0 mM to 500 mM NaCl in Buffer A over 5 column volumes with elution monitored at 280 nm and 488 nm. Pooled fractions were buffer exchanged into fresh Buffer A supplemented with 150 mM NaCl (Buffer B) with Amicon® Ultra centrifugal concentrators. Buffer exchanged protein samples were applied to a SP Superdex™ 200 gel filtration column (GE Healthcare) with elution monitored at 280 nm and 488 nm. The purified protein sample was finally stored in Buffer B. Protein concentration was determined with the DC Protein assay kit (Bio-Rad) using bovine serum albumin (BSA) as a protein standard. The assay was performed as to the manufactures guidelines for use in a microplate assay.

Size exclusion chromatography

Gel filtration standards (Biorad) were applied to a Superdex™ 75 column (20 ml bed volume, 0.5 ml/min flow rate). As per the manufacturers guidelines with protein elution monitored at 280 nm. A standard curve was generated from the plot LogMw against K_{av}, where $K_{av} = (V_e - V_o)/(V_t - V_o)$, V_e is the elution volume, V_t is the total volume and V_o is the void volume. Protein samples were prepared in Buffer B to final concentrations of 25, 50 or 100 uM and applied to a Superdex™ 75 column with protein elution monitored by absorbance at 488 nm. Elution volumes were determined for each sample and K_{av} values calculated. Using the standard curve estimated molecular weights could be determined for each protein sample.

Fit to 2 state unfolding.

Equilibrium unfolding was fit to a 2-state model in the GraphPad Prism software (*equation 1*) to estimate approach to equilibrium (see Supporting Methods).

$$Y_N = \alpha_N + \beta_N[\mathrm{D}],\ \ Y_D = \alpha_D + \beta_D[\mathrm{D}]$$

$$F = Y_N - (Y_N - Y_D)\frac{exp\left(\frac{m_{N-D}([D]-[D]_{50\%})}{RT}\right)}{1+exp\left(\frac{m_{N-D}([D]-[D]_{50\%})}{RT}\right)} \qquad \textit{equation 1}$$

Where F is the fraction of folded protein, Y_N and Y_D are intensities of native and denatured states, respectively. To take into account sloping baselines for the fluorescence data, Y_N and Y_D are described as a function of α_N, β_N, α_D and β_D, respectively. Where α_N and α_D are the fluorescence intensities of the native and denatured states, respectively, and β_N and β_D are the slopes of the native and denatured baselines. $m_{N\text{-}D}$ is a constant that describes the dependence of ΔG on denaturant concentration, [D], between the native and denatured states. $[D]_{50\%}$ is the estimated midpoint of the unfolding transition and represents the concentration of denaturant at which 50% of the protein is folded and 50% is unfolded.

Supporting Figures.

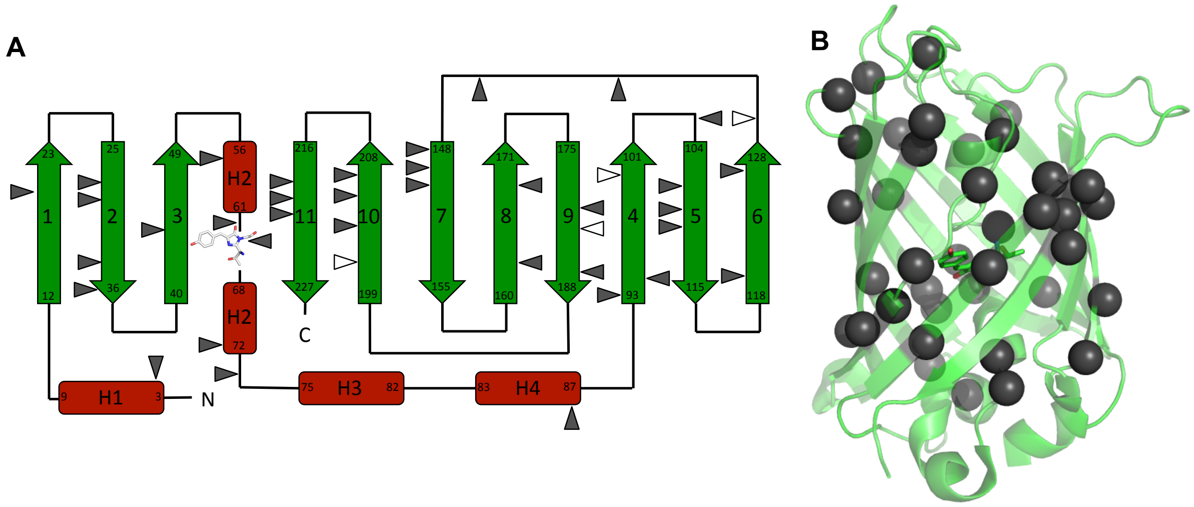

Supporting Figure S1, related to Figure 1. Mapping non-tolerated single amino acid deletion mutations with respect to EGFP (A) secondary and (B) tertiary structure. (A). The secondary structure arrangement and overall topology of EGFP shows the arrangement of β-strands (green), α-helices (red) and loops (black). Disruptive single amino acid deletions identified in this study are indicated by black triangles and trinucleotide deletions generating stop codon are shown as white triangles. (B) Map of single amino acid deletions onto the tertiary structure of EGFP. Cartoon representation of EGFP (green) with disruptive deletions indicated by black spheres.

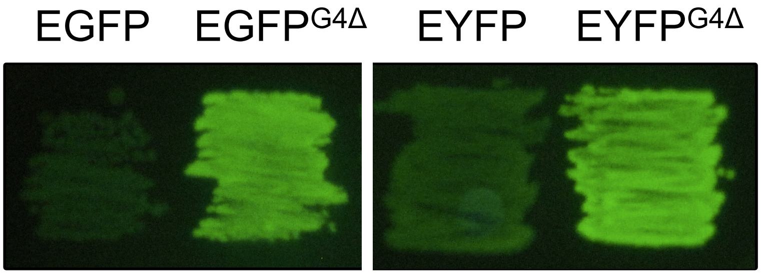

Supporting Figure S2, related to Figure 3. Colour version of cellular fluorescence of the EGFP and EYFP, and the corresponding G4Δ variants presented in Figure 3 in the main manuscript.

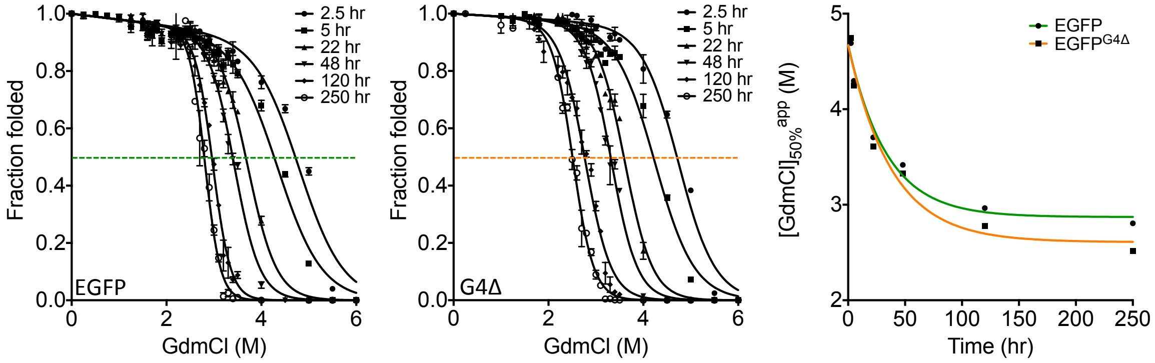

Supporting Figure S3, related to Figure 4 and Table 1. Guanidinium chloride induced equilibrium unfolding and equilibrium kinetics. Fluorescence emission at 520 nm after excitation at 480 nm was monitored for (A) EGFP and (B) EGFP$^{G4\Delta}$, over 250 hrs (as indicated in the figures) and data were fit to a two state model (GraphPad Prism). **C,** Apparent $[GdmCl]_{50\%}$ values (the [GdmCl] at which 50% of the samples are in the native and 50% in the denatured states) were plot against time and fit to single exponential decay curves to assure close approach to equilibrium.

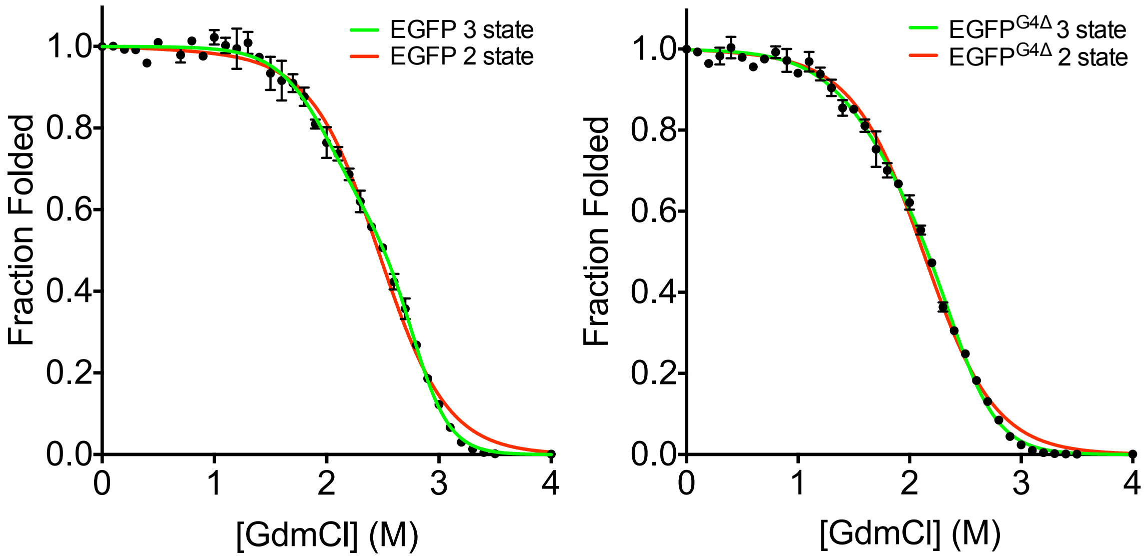

Supporting Figure S4, related to Figure 4. Two state and three state model fits to equilibrium unfolding data. Equilibrium unfolding data for EGFP (left panel) and EGFP$^{G4\Delta}$ (right panel) fit to a two state (red) or three state (green) model highlights the poor fit of the data to a two state model.

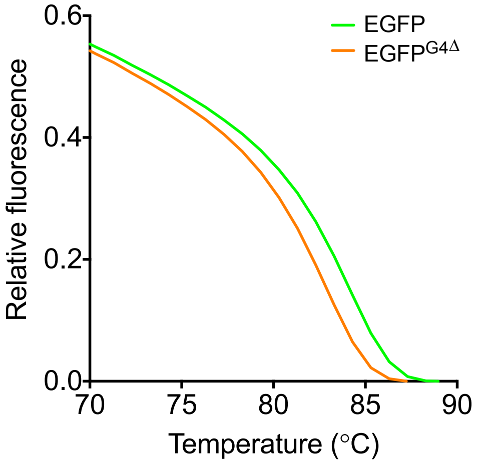

Supporting Figure S5, related to Figure 4. Thermal melting curves for EGFP and EGFP$^{g4\Delta}$. Melting temperatures (T*m*) of EGFP and EGFP$^{G4\Delta}$ were determined by monitoring fluorescence with an Opticon 2 qPCR thermal cycler (MJ Research) while ramping the temperature from 25-98°C. Protein samples were diluted to a final concentration of 1 µM in 50 mM sodium phosphate buffer pH 8.0 (total volume 50 µl) and the temperature ramped at 1°C/min. MJ Research Software supplied with the qPCR machine was used to determined an apparent melting temperature.

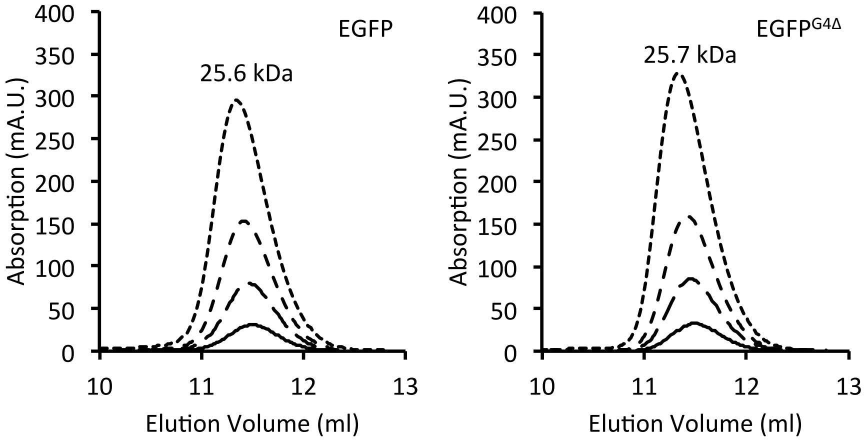

Supporting Figure S6, related to Figure 5 and Table 3. Size exclusion chromatography of EGFP$^{G4\Delta}$. The elution profiles of (A) EGFP and (B) EGFP$^{G4\Delta}$ at 10 µM (black line), 25 µM (long dash), 50 µM (medium dash) and 100 µM (short dash). The estimated molecular weight based on the peak elution volume is shown on the graph.

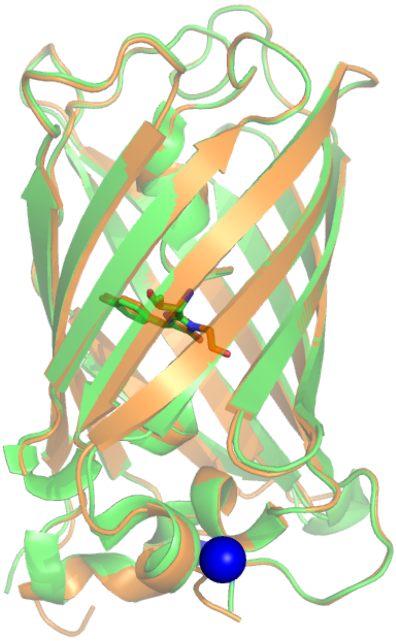

Supporting Figure S7, related to Figure 5. Overlap of EGFP (green) with $EGFP^{G4Δ}$ (orange) with the G4 residue in EGFP highlighted as a blue sphere and the chromophore shown as stick representation. The RMSDs between the two structures in terms of backbone and all atoms was 0.6Å and 1.2Å respectively.

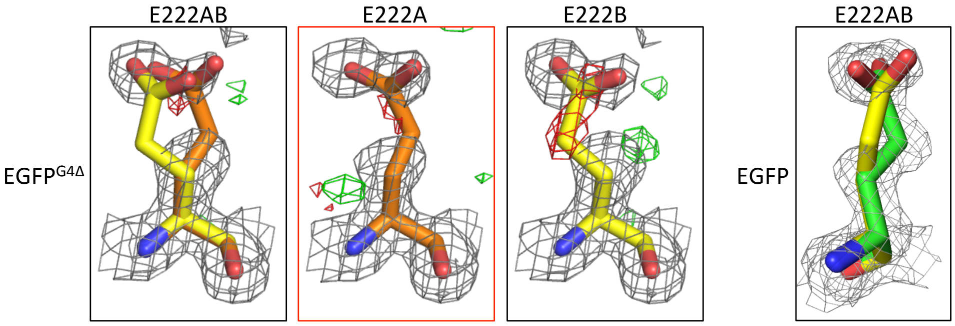

Supporting Figure S8, related to Figure 5. Rationale behind modelling of E222 as a single conformer in $EGFP^{G4Δ}$. Modelling of residue E222 as either the single conformer A (E222A), the single conformer B (E222B) or as a double conformer (E222AB). The electron density does not fully support the modelling of E222 in $EGFP^{G4Δ}$ as a double conformer. The model used in final crystal structure refinement is highlighted in the red box (E222A).

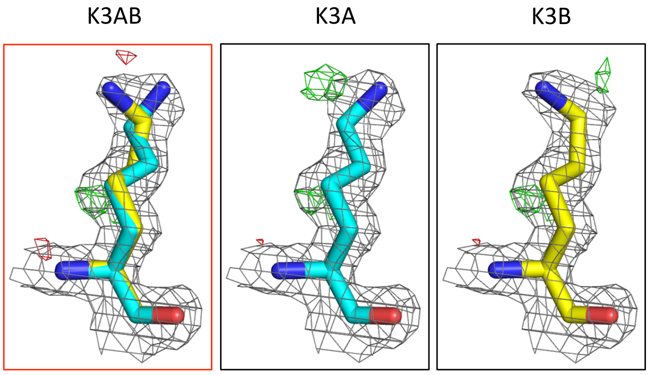

Supporting Figure S9, related to Figure 5. Rationale behind modelling of K3 as a double conformer in EGFP$^{G4\Delta}$. Modelling of residue K3 as either the single conformer A (K3A) or conformer B (K3B) does not fully satisfy the electron density. Modelling of residue K3 by both conformers does satisfy the electron density. The model used in final crystal structure refinement is highlighted in a red (K3AB) box.

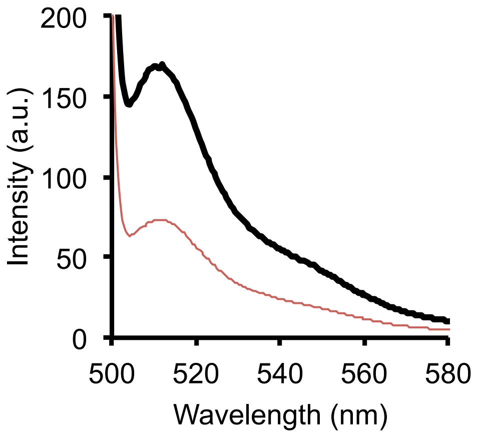

Supporting Figure S10, related to Figure 3. Whole cell fluorescence emission (excited at 488 nm) spectra for cultures grown at 37°C expressing EGFP (black line) or EGFP$^{K3N\text{-}G4\Delta}$. Cell cultures were standardised to an OD_{600} of 0.1.

Supporting Table S1, related to Figure 1. Tolerated TNDs in *egfp* and subsequent amino acid mutations

Nucleotide deletion[a]	Amino acid Mutation[b]	Frequency	Secondary structure[c]	SASA (Å^2)	% SASA
$_{3}$G**TG A**GC$_{10}$	V1Δ S2G	2	N-terminus	ND	ND
$_{9}$AA**G GG**C$_{16}$	K3N G4Δ	4	H1	2.77	13
$_{12}$G**GC G**AG$_{19}$	G4Δ	8	H1	2.77	13
$_{12}$GG**C GA**G$_{19}$	E5Δ	2	H1	57.09	42
$_{18}$**GAG**$_{22}$	E6Δ	1	H1	84.96	42
$_{27}$A**CC G**GG$_{34}$	T9Δ G10R	6	H1	102.95	70
$_{27}$AC**C GG**G$_{34}$	G10Δ	2	Loop H1-S1	37.91	38
$_{36}$**GTG**$_{40}$	V12Δ	1	S1	9.20	12
$_{75}$**CAC**$_{79}$	H25Δ	2	S2	79.74	54
$_{114}$**ACC**$_{118}$	T38Δ	3	Loop S2-S3	65.55	37
$_{144}$**TGC**$_{148}$	C48Δ	1	S3	3.92	9
$_{147}$**ACC**$_{151}$	T50Δ	1	Loop S3-H2	81.50	50
$_{150}$A**CC G**GC$_{157}$	T50Δ G51S	2	Loop S3-H2	81.50	50
$_{159}$C**TG C**CC$_{166}$	L53Δ	1	Loop S3-H2	2.58	11
$_{225}$C**CC G**AC$_{232}$	P75Δ D76H	2	H3	21.59	17
$_{225}$**GAC**$_{229}$	D76Δ	2	H3	118.40	73
$_{237}$**AAG**$_{241}$	K79Δ	1	H3	58.66	24
$_{396}$GA**G GA**C$_{403}$	E132D D133Δ	1	Loop S6-S7	108.24	72
$_{411}$**GGG**$_{415}$	G138Δ	2	Loop S6-S7	26.72	21
$_{459}$A**TG G**CC$_{466}$	M153Δ A154T	2	S7	69.42	37
$_{462}$G**CC G**AC$_{469}$	A154Δ	5	S7	30.50	23
$_{465}$**GAC**$_{469}$	D155Δ	4	S7	22.16	22
$_{474}$AA**G AA**C$_{481}$	K158Δ	1	Loop S7-S8	106.96	57
$_{480}$**GGC**$_{484}$	G160Δ	1	S8	11.54	10
$_{513}$AT**C GA**G$_{520}$	I171M E172Δ	3	Loop S8-S9	88.73	39
$_{522}$**GGC**$_{526}$	G174Δ	2	Loop S8-S9	68.18	52
$_{525}$**AGC**$_{529}$	S175Δ	1	Loop S8-S9	59.04	34
$_{567}$G**GC G**AC$_{574}$	G189Δ	1	Loop S9-S10	22.96	36
$_{570}$G**AC G**GC$_{577}$	D190Δ	1	Loop S9-S10	152.83	100
$_{576}$C**CC G**TG$_{583}$	P192Δ V193L	3	Loop S9-S10	130.44	95
$_{588}$**CCC**$_{592}$	P196Δ	1	Loop S9-S10	5.11	16
$_{591}$**GAC**$_{595}$	D197Δ	1	Loop S9-S10	54.34	62
$_{594}$**AAC**$_{598}$	N198 Δ	1	Loop S9-S10	100.68	71
$_{633}$C**CC A**AC$_{640}$	P211Δ N212H	3	Loop S10-S11	112.07	58
$_{678}$G**CC GCC G**GG$_{687}$	A226Δ A227Δ	1	S11	30.10 / 28.62	12 / 20
$_{681}$G**CC G**GG$_{688}$	A227Δ	5	S11	28.62	20
$_{681}$GC**C GG**G$_{688}$	G228Δ	2	C-terminus	48.44	38
$_{690}$AC**T CT**C$_{697}$	L231Δ	1	C-terminus	178.68	93
$_{699}$A**TG G**AC$_{706}$	M233Δ D234N	2	C-terminus	ND	ND
$_{702}$GA**C GA**G$_{709}$	D234E E235Δ	2	C-terminus	ND	ND
$_{705}$**GAG**$_{709}$	E235Δ	1	C-terminus	ND	ND
$_{711}$**TAC**$_{715}$	Y237Δ	1	C-terminus	ND	ND

[a] Numbers refer to gene sequence numbering for *egfp* (GFPmut1)

[b] Δ after a residue number signifies that residue has been deleted, protein numbering as per wtGFP

[c] Secondary structure elements as defined by Fig 1, helices (H), strands (S).

Supporting Table S2, related to Figure 1. Non-tolerated TNDs in *egfp* and subsequent amino acid mutations

Nucleotide deletion[a]	Amino acid Mutation[b]	Frequency	Secondary structure[c]	SASA (Å^2)
$_{9}$AAG GGC$_{16}$	K3Δ G4S	1	H1	178.25
$_{60}$GGC GAC$_{67}$	G20Δ	3	S1	5.93
$_{81}$TTC AGC$_{88}$	F27Δ S28C	1	S2	5.40
$_{90}$TCC GGC$_{97}$	S30Δ G31C	3	S2	31.28
$_{99}$GGC GAG$_{106}$	E34Δ	2	S2	89.14
$_{105}$GGC GAT$_{112}$	D36Δ	1	S2	26.72
$_{135}$AAG TTC$_{142}$	K45Δ F46I	1	S3	45.42
$_{168}$CCC TGG$_{175}$	W57Δ	1	H2	12.84
$_{171}$TGG$_{174}$	W57Δ	3	H2	12.84
$_{189}$ACC CTG$_{196}$	L64Δ	1	Loop H2-H3	0.00
$_{192}$CTG ACC$_{199}$	L64Δ T65P	2	Loop H2-H3/Cro	0.00
$_{198}$TAC GGC$_{205}$	Y66Δ G67C	1	Cro	ND
$_{216}$AGC$_{220}$	S72Δ	1	H3	2.38
$_{219}$CGC$_{223}$	R73Δ	1	Loop H3-H4	87.13
$_{261}$GCC$_{265}$	A87Δ	2	H5	5.30
$_{279}$GTC CAG$_{286}$	V93Δ Q94E	1	S4	19.40
$_{282}$CAG$_{286}$	Q94Δ	1	S4	5.31
$_{300}$TTC AAG$_{307}$	F100Δ K101STOP	1	S4	3.91
$_{309}$GAC GGC$_{316}$	D103Δ	1	Loop S4-S5	28.42
$_{321}$AAG ACC$_{328}$	K107Δ	1	S5	98.33
$_{330}$GCC GAG$_{337}$	A110Δ	3	S5	5.59
$_{330}$GCC GAG$_{337}$	E111Δ	1	S5	53.03
$_{360}$GTG$_{364}$	V120Δ	3	S6	8.67
$_{360}$GTG AAC$_{367}$	V120Δ N121D	1	S6	8.67
$_{381}$GGC ATC$_{388}$	G127Δ I128V	1	S6	0.42
$_{390}$TTC AAG$_{397}$	F130Δ K131STOP	1	Loop S6-S7	10.87
$_{411}$CTG$_{415}$	L137Δ	1	Loop S6-S7	22.36
$_{435}$TAC$_{439}$	Y145Δ	1	Loop S6-S7	23.93
$_{444}$CAC$_{448}$	H148Δ	3	S7	9.18
$_{450}$GTC TAT$_{457}$	V150Δ Y151D	3	S7	0.01
$_{450}$GTC TAT$_{457}$	Y151Δ	2	S7	103.92
$_{486}$AAG$_{490}$	K162Δ	1	S8	64.53
$_{507}$CAC$_{511}$	H169Δ	3	S8	8.20
$_{510}$AAC ATC$_{516}$	N170Δ	1	S8	50.12
$_{540}$GAC$_{544}$	D180Δ	1	S9	44.22
$_{546}$TAC CAG$_{553}$	Y182STOP Q183Δ	2	S9	0.00
$_{561}$CCC$_{565}$	P187Δ	1	S9	17.65
$_{600}$TAC CTG$_{607}$	Y200STOP L201Δ	1	S10	0.55
$_{609}$ACC CAG$_{616}$	Q204Δ	1	S10	101.34
$_{615}$TCC GCC$_{622}$	A206Δ	1	S10	55.05
$_{618}$GCC CTG$_{625}$	L207Δ	1	S10	22.63
$_{621}$CTG AGC$_{628}$	L207Δ S208R	1	S10	22.63
$_{654}$ATG GTC$_{661}$	M218I V219Δ	1	S11	27.30
$_{660}$CTG$_{664}$	L220Δ	1	S11	0.00
$_{663}$CTG$_{667}$	L221Δ	1	S11	65.26

[a] Numbers refer to gene sequence numbering for *egfp* (GFPmut1)
[b] Δ after a residue number signifies that residue has been deleted, protein numbering as per wtGFP
[c] Secondary structure elements as defined by Fig 1, helices (H), strands (S).

Supporting References

Baldwin, A.J., Arpino, J.A., Edwards, W.R., Tippmann, E.M., and Jones, D.D. (2009). Expanded chemical diversity sampling through whole protein evolution. Molecular BioSystems *5*, 764-766.
